# Estimating uncertainty in read‐out patterns: Application to controls‐based denoising and voxel‐based morphometry patterns in neurodegenerative and neuropsychiatric diseases

**DOI:** 10.1002/hbm.26246

**Published:** 2023-03-22

**Authors:** Dominik Blum, Tobias Hepp, Valdimir Belov, Roberto Goya‐Maldonado, Christian la Fougère, Matthias Reimold

**Affiliations:** ^1^ Department of Nuclear Medicine and Clinical Molecular Imaging University Hospital Tübingen Tübingen Germany; ^2^ Department of Radiology University Hospital Tübingen Tübingen Germany; ^3^ Max Planck Institute for Intelligent Systems Tübingen Germany; ^4^ Laboratory of Systems Neuroscience and Imaging in Psychiatry University Medical Center Göttingen Göttingen Germany

**Keywords:** controls‐based denoising, pattern quantification, pattern uncertainty

## Abstract

Quantifying pathology‐related patterns in patient data with pattern expression score (PES) is a standard approach in medical image analysis. In order to estimate the PES error, we here propose to express the uncertainty contained in read‐out patterns in terms of the expected squared Euclidean distance between the read‐out pattern and the unknown “true” pattern (squared standard error of the read‐out pattern, SE^2^). Using SE^2^, we predicted and optimized the net benefit (NBe) of the recently suggested method controls‐based denoising (CODE) by weighting patterns of nonpathological variance (NPV). Multi‐center MRI (1192 patients with various neurodegenerative and neuropsychiatric diseases, 1832 healthy controls) were analysed with voxel‐based morphometry. For each pathology, accounting for SE^2^, NBe correctly predicted classification improvement and allowed to optimize NPV pattern weights. Using these weights, CODE improved classification performances in all but one analyses, for example, for prediction of conversion to Alzheimer's disease (AUC 0.81 vs. 0.75, *p* = .01), diagnosis of autism (AUC 0.66 vs. 0.60, *p* < .001), and of major depressive disorder (AUC 0.62 vs. 0.50, *p* = .03). We conclude that the degree of uncertainty in a read‐out pattern should generally be reported in PES‐based analyses and suggest using weighted CODE as a complement to PES‐based analyses.

## Graphical abstract

We describe the level of uncertainty in read‐out patterns from brain image data using the concept of a squared standard error and estimate the error in pattern expression scores. By optimizing weights of nonpathological variance patterns, we predict classification improvement by our method in the most common neurodegenerative and neuropsychiatric disorders.

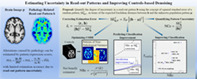

## 1 INTRODUCTION

While powerful algorithms are continuously improving medical image analysis, their application in clinical practice remains aggravated due to limited availability of training data (Castro et al., 2020). A simple image analysis approach, well‐established for clinical applications, is to calculate a pathology‐related pattern in terms of a group difference from training data and to calculate a pattern expression score (PES) for each subject (Blazhenets et al., 2020; Blazhenets et al., 2021; Meles et al., 2018; Oh et al., 2014; Sörensen et al., 2020). Blum et al. recently showed that PES‐based analysis can be improved by using information (patterns of nonpathological variance) from (easily available) multi‐center controls. Controls‐based denoising, CODE (Blum et al. (2019)), yielding a classification performance comparable to standard machine learning methods.

Predictions from limited training samples require estimation of model uncertainties. In order to quantify the degree of uncertainty in a read‐out pattern, we propose the concept of squared standard error of a random pattern, ${\mathrm{SE}}_{b}^{2},$ considering the Euclidean distance between the read‐out pattern and the unknown population mean. In this article, we derive an uncertainty‐corrected estimation of the error ϵ of PES when describing the degree of alterations caused by pathology.

In order to estimate the net benefit of CODE (net benefit estimator, NBe; the ratio by which the signal‐to‐noise ratio of the PES improves), ϵ before and after denoising has to be estimated. We here evaluate ${\mathrm{SE}}_{b}^{2}$ for improving estimation of NBe and for optimization of CODE. Blum et al. presented CODE in a basic form, that is, NPV patterns were completely removed. In this article, we evaluate optimized denoising by including weights that determine the degree to which NPV patterns are removed.

For evaluation, we applied our method to structural brain‐MRI from 1192 patients and 1832 healthy controls, analyzed with voxel‐based morphometry (VBM, voxel‐wise gray‐matter probability (Ashburner & Friston, 2000; Scarpazza & Simone, 2016)), in the most common neurodegenerative and neuropsychiatric diseases, that is, Alzheimer's disease (AD), mild cognitive impairment (MCI), Parkinson's disease (PD), autism (AUT), schizophrenia (SCZ), and major depressive disorder (MDD). Typical VBM patterns have been reported e.g. in AD (Toniolo et al., 2018), SCZ (Torres et al., 2016), PD (Solana‐Lavalle & Rosas‐Romero, 2021), and AUT (Riddle et al., 2017). In healthy subjects, VBM reveals patterns associated with, for example, age, sex, and cognitive performance (Bourisly et al., 2015; Ramanoël et al., 2018; Schmitt et al., 2020).

## 2 MATERIALS AND METHODS

### 2.1 Mathematical notation

Scalars are denoted by nonbolded uppercase letters (e.g. PSS). Lower case bold letters denote vectors, that is, single voxels (*I* × 1 vector with *I* subjects) and image data (1 × *J* vector with *J* voxels). *N* images are stored in a *N* × *P*—matrix, denoted by bold uppercase letters. ${\text{proj}}_{x}$ denotes the orthogonal projection onto x. Image data and PES after denoising is denoted by superscript d. Transpose is denoted by uppercase *T*, standard deviation by *σ*, dot product by $\left\langle \cdot ,\cdot \right\rangle$ and L2‐norm by ‖⋅‖. Table 1 provides a list of abbreviations used in the theory section.

**TABLE 1:** Variables introduced in the theory section.

Related to linear pathology model	${\mathrm{PSS}}_{i}$	Pathology‐specific score of patient i	Degree of alterations caused by pathology	Equation (1)
	μ	Pathology‐related pattern	Pattern of alterations caused by pathology	Equation (1)
Related to read‐out pattern	b	Read‐out pattern	Estimate of μ	Equation (A6)
	${\mathrm{PES}}_{i}$	Pattern expression score of patient i	Degree to which a read‐out pattern **b** is present in individual image data	Equation (2)
	${\mathrm{SE}}_{b}^{2}$	Squared standard error of read‐out pattern	Proposed measure of read‐out pattern uncertainty	Equations (A9) and (3)
	NCe	Noise component estimator	Normalized ${\mathrm{SE}}_{b}^{2}$	Equation (4)
Related to CODE	NRe	Noise reduction estimator	Degree to which noise is reduced by CODE (in %)	Equation (11)
	SRe	Signal reduction estimator	Degree to which signal is reduced by CODE (in %)	Equation (12)
	NBe	Net benefit estimator	Net benefit of CODE (factor by which signal‐to‐noise ratio of PES improves)	Equations (9) and (10)

### 2.2 Theory

#### 2.2.1 Linear pathology model, pattern expression score, and uncertainty in the read‐out pattern

This section summarizes some basic concepts of this article. A more detailed, mathematical description is given in the Appendix A.

Quantification of pathology‐related alterations in terms of a so‐called pattern expression score, PES, is based on a linear pathology model, in which the image of patient i is comprised of a pathology‐related component and a residual component. The pathology related component is the product of a particular pathology‐related pattern **μ** and the degree to which this pattern is present due to an underlying pathological process. Here, this degree is denoted pathology specific score PSS_i_. The residual component $r_{i}$ contains patterns of physiological variance (related, e.g., to age, sex, education) and variance due to the image generating process itself (e.g., imaging device, protocol).

(1) $$p_{i}={\mathrm{PSS}}_{i}\;\mu +\;r_{i}.$$

Of note, mathematically speaking, **μ** and $r_{i}$ are not orthogonal, corresponding to the fact that ${\mathrm{PSS}}_{i}\mu$ covers only those changes that are caused by pathology while some similarity between **μ** and $r_{i}$ cannot be ruled out.

The PES of **μ** is defined as the normalized dot product between $p_{i}$ and **μ** (Equation A1). By multiplying both sides of Equation 1 with $\mu /∥\mu {∥}^{2}$, one can see that ${\mathrm{PES}}_{\mu ,i}$ can be used to estimate ${\mathrm{PSS}}_{i}$. The error associated with estimating PSS_i_ depends on the degree of similarity between **μ** and $r_{i}$ (Equation A3).

In this article, we explore the fact that the true pattern **μ** is unknown and that an estimate b from a limited sample of patients and controls has to be used instead. Since b is used to estimate ${\mathrm{PSS}}_{i}$, we here refer to it as *read‐out pattern* and, unless specified otherwise, PES refers to the normalized dot product between p and b:

(2) $${\mathrm{PES}}_{i}=\frac{\left\langle p_{i},b\right\rangle }{{\left\|b\right\|}^{2}}$$

When distinguishing between b and **μ**, the error from estimating PSS_i_ with PES, $\epsilon_{i}$ (Equation A5), contains the denominator $\left\langle \mu ,b\right\rangle$, which corresponds to the fact that, the more b deviates from **μ**, the larger the error becomes. Since **μ** is unknown, $\left\langle \mu ,b\right\rangle$ cannot be calculated. However, as shown below and in the Appendix A, it can be estimated. To this end, $\left\langle \mu ,b\right\rangle$ is expressed in terms of the squared Euclidean distance between b and **μ** (Equation A7).

In this article, we calculate b as mean difference between patients P and healthy controls H. As shown in the Appendix A, if defining the deviation between two images in terms of their Euclidean distance (i.e., $d=\sqrt{d_{1}^{2}+d_{2}^{2}+\cdots }$), the expected squared deviation of b from **μ** can be calculated the same way as if, instead of images, we were dealing with a scalar random variable. Therefore, we denote it the squared standard error of b, ${\mathrm{SE}}_{b}^{2}$.

As shown in the Appendix A, ${\mathrm{SE}}_{b}^{2}$ can be expressed as a function of the variances in each voxel j across P and H:

(3) $${\mathrm{SE}}_{b}^{2}=\frac{1}{N_{P}}\sum_{j=1}^{J}\mathrm{Var}\left(P_{j}\right)+\frac{1}{N_{H}}\sum_{j=1}^{J}\mathrm{Var}\left(H_{j}\right)$$

with $N_{P}$ being the number of patients and $N_{H}$ being the number of controls.

To make ${\mathrm{SE}}_{b}^{2}$ independent from intensity scaling of the voxel data and to allow for comparison across different read‐out patterns and different imaging modalities, we normalize ${\mathrm{SE}}_{b}^{2}$ by ${\left\|b\right\|}^{2}$. We call the normalized ${\mathrm{SE}}_{b}^{2}$ noise component estimator, NCe,

(4) $$\mathrm{NCe}=\frac{{\mathrm{SE}}_{b}^{2}}{{\left\|b\right\|}^{2}}.$$

According to Equations (A5), (A8), and (4), the error from estimating ${\mathrm{PSS}}_{i}$ with PES, $\epsilon_{i}$, can then be estimated by

(5) $${\hat{\epsilon }}_{i}=\frac{\left\langle r_{i},b\right\rangle }{\left\langle \mu ,b\right\rangle }=\frac{\left\langle r_{i},b\right\rangle }{\left(1-\mathrm{NCe}\right){\left\|b\right\|}^{2}}$$

#### 2.2.2 Standard CODE

Before calculating the PES, Blum et al. (2019) suggested obtaining NPV patterns C from a separate sample of controls using PCA (Jolliffe, 2002) and removing them from image data

(6) $$p_{i}^{d}=p_{i}-{\text{proj}}_{C}\left(p_{i}\right)=p_{i}-p_{i}C^{⊺}C.$$

To reduce computational time, the denoised PES can be calculated by removing NPV patterns from the read‐out pattern instead from image data, ${\mathrm{pes}}^{d}=\left\langle p_{i}^{d},b\right\rangle =\left\langle p_{i},b^{d}\right\rangle$. According to (5) and making use of $\left\langle b,b^{d}\right\rangle ={\left\|b^{d}\right\|}^{2}$, the new estimation error is given by

(7) $${\hat{\epsilon }}_{i}^{d}=\frac{\left\langle r_{i},b^{d}\right\rangle }{\left(1-{\mathrm{NCe}}^{d}\right){\left\|b^{d}\right\|}^{2}}.$$

**FIGURE 1:**
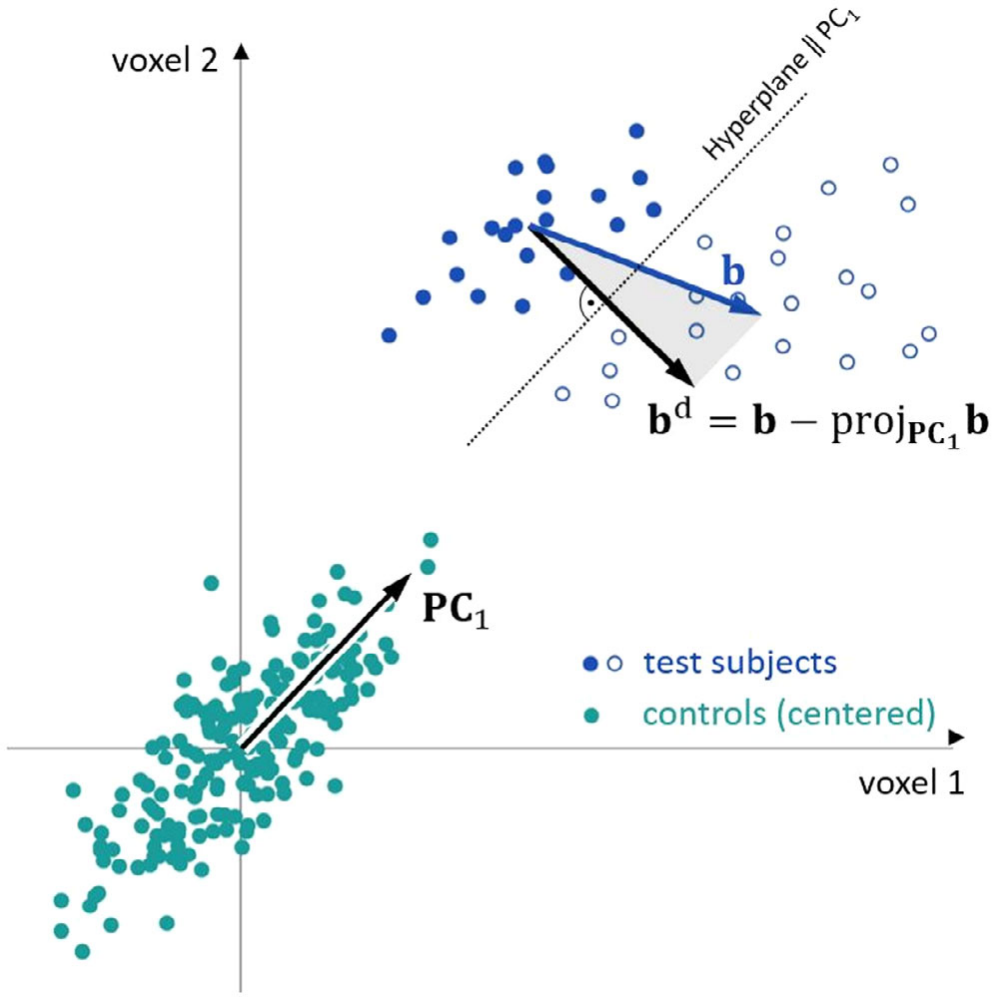
Illustration of the benefit of denoising for two voxels. Removing PC 1 (NPV pattern 1) from the difference of means b results in a new pattern $b^{d}$, which allows for improved classification.

Figure 1 shows the benefit of denoising schematically for two voxels. The two groups of test subjects (blue dots and circles) cannot be perfectly separated with b, the difference of means. Removing PC 1 (NPV pattern 1) from b results in a new pattern $b^{d}$, which allows for improved classification.

#### 2.2.3 Net benefit estimator

We define the ratio of $\sigma \left(\varepsilon \right)$ before and after CODE as net benefit of denoising

(8) $$\mathrm{Net}\;\text{benefit}=\frac{\sigma \left(\varepsilon \right)}{\sigma \left(\varepsilon^{d}\right)}.$$

Estimating the net benefit according to (5) and (7) yields the net benefit estimator (NBe),

(9) $$\mathrm{NBe}=\frac{\sigma \left(\hat{\varepsilon }\right)}{\sigma \left({\hat{\varepsilon }}^{d}\right)}=\frac{\left(1-{\mathrm{NCe}}^{d}\right){\left\|b^{d}\right\|}^{2}}{\left(1-\mathrm{NCe}\right){\left\|b\right\|}^{2}}\cdot \frac{\sigma \left({\mathrm{Rb}}^{⊺}\right)}{\sigma \left({\mathrm{Rb}}^{d⊺}\right)}.$$

Denoising has effects on both noise and signal components in the data. To facilitate interpretation of denoising effects, we propose an alternative notation of NBe:

(10) $$\mathrm{NBe}=\frac{1-\mathrm{SRe}}{1-\mathrm{NRe}}$$

with NRe denoting the estimated noise reduction

(11) $$\mathrm{NRe}=1-\frac{\sigma \left({\mathrm{Rb}}^{d,T}\right)}{\sigma \left({\mathrm{Rb}}^{T}\right)}$$

and SRe denoting the associated signal reduction

(12) $$\mathrm{SRe}=1-\frac{\left(1-{\mathrm{NCe}}^{d}\right){\left\|b^{d}\right\|}^{2}}{\left(1-\mathrm{NCe}\right){\left\|b\right\|}^{2}},$$

Here, both NCe and SRe are reported in [%].

#### 2.2.4 Weighted denoising

So far, denoising refers to completely removing NPV patterns. It is conceivable that CODE is more effective (higher net benefit) when patterns are only partially removed, for example, $b_{w}^{d}=b-w_{1}c_{1}^{T}c_{1}$ for denoising with PC 1, where weight(s) *w* can be determined via optimization. Denoising with an optimized weight furthermore ensures that CODE does not worsen the classification (*w* = 0 corresponds to not performing CODE).

In preliminary analyses (data not published), optimization of each individual PC‐weight *w*_*k*_ yielded results that were not generalizable. Therefore, we evaluate the simplified approach of applying the same weight *w* for all PCs.

Standard CODE (Section 2.2.2) corresponds to denoising with *w* = 1. In standard CODE, it holds that $\left\langle b,b_{w}^{d}\right\rangle =\left\langle b_{w}^{d},b_{w}^{d}\right\rangle ={\left\|b_{w}^{d}\right\|}^{2}$ while, for w≠1, $\left\langle b,b_{w}^{d}\right\rangle ={\left\|b_{v}^{d}\right\|}^{2}$ with *v* and *w* being connected via $1-v={\left(1-w\right)}^{2}$. The optimization problem of weighted denoising is thus

(13) $$\begin{array}{l} w^{*}={\text{argmin}}_{w}\sigma \left({\hat{\varepsilon }}^{d}\right)={\text{argmin}}_{w}\frac{\sigma \left({{\mathrm{Rb}}_{w}^{d}}^{⊺}\right)}{\left(1-{\mathrm{NCe}}_{v}^{d}\right){\left\|b_{v}^{d}\right\|}^{2}}\\ \text{subject}\;\text{to}\;{\mathrm{NCe}}_{v}^{d}<1. \end{array}$$

Of note, we have shown that under the assumption of ${\mathrm{NCe}}_{v}^{d}=0$, there exists a closed‐form solution, even when allowing for different PC weights. The derivation of the solution can be found in supplementary material.

Source codes (MATLAB) for the calculation of NCe and the relevant metrics related to CODE are available through MathWorks Exchange Servers.

### 2.3 Demographics and clinical data

We collected 3024 multi‐center brain‐MRI scans (T1‐weighted, 3 Tesla) from the freely accessible image databases ADNI ((Petersen et al., 2010), http://adni.loni.usc.edu/), AIBL ((Ellis et al., 2009), https://aibl.csiro.au), ABIDE ((Di Martino et al., 2014), https://fcon_1000.projects.nitrc.org/indi/abide/), PPMI ((Initiative, 2011), https://www.ppmi-info.org), IXI ((BioMed, n.d.), https://brain-development.org/ixi-dataset), COBRE ((Aine et al., 2017); (Wang et al., 2016), http://fcon_1000.projects.nitrc.org/indi/retro/cobre.html), FBIRN (Keator et al. (2016); Wang et al. (2016), https://www.nitrc.org/projects/fbirn) and from the Laboratory of Systems Neuroscience and Imaging in Psychiatry (SNIP‐Lab, www.sniplab.uni-goettingen.de, University Medical Center Göttingen, UMG). The data set comprises 1832 MRI scans from HC and 1192 scans from patients diagnosed with one of the following neurodegenerative or neuropsychiatric disease: AD, MCI, PD, AUT, SCZ, and MDD. Of the MCI patients, 151 converted to AD (MCIc) within 6 years and 90 remained stable (MCIs) within follow‐up (>6 years). For PD analysis, we included the MoCA score (Sammer & Lenz, 2020), which reflects the degree of cognitive impairment. Demographic data are summarized in Table 2.

**TABLE 2:** Demographic data.

					Associated PCA sample (multi‐center, loosely age matched)	
Database	Groups	Sample size	Age (mean ± SD)	Sex (m/f)	Sample size	Age (mean ± SD)
ADNI	HC	325	73 ± 7	44/56	643	69 ± 9
	AD	205	75 ± 9	55/45		
	MCIs	90	72 ± 7	62/38		
	MCIc	151	74 ± 7	60/40		
ABIDE	HC	569	17 ± 7	83/17	621	28 ± 14
	AUT	317	16 ± 7	90/10		
AIBL	HC	415	73 ± 6	41/59	—	—
COBRE and FBIRN	HC	149	38 ± 12	66/34	1030	44 ± 22
	SCZ	128	38 ± 13	77/23		
IXI	HC	181	47 ± 17	48/52	—	—
PPMI	HC	114	60 ± 11	66/34	1127	63 ± 17
	PD	234	61 ± 9	64/36		
UMG	HC	79	34 ± 14	53/47	1100	44 ± 21
	MDD	67	37 ± 15	54/46		

*Note*: HC subjects for PCA were obtained from all databases but the one that was used for analysis.

Abbreviations: AD, Alzheimer's disease; AUT, autism; HC, healthy controls; MCI, mild cognitive impairment; MCIc, conversion to AD within 6 years; MCIs, stable, no conversion to AD during follow‐up (≥6 years); MDD, major depressive disorder; PD, Parkinson's disease; SCZ, schizophrenia.

### 2.4 Voxel‐based morphometry

For VBM analysis, we used the Computational Anatomy Toolbox (CAT 12.7 (Gaser & Dahnke, 2022)), a comprehensive toolbox for analysis of structural MRI data. Initial processing comprised denoising, bias‐correction, affine‐registration, and unified segmentation (Ashburner & Friston, 2005), followed by a refined processing comprising skull‐stripping, local white matter hyperintensity detection, local intensity transformation, adaptive maximum a posteriori segmentation, partial volume estimation as well as spatial normalization and total intracranial volume (TIV) estimation. For both initial processing and refined processing parameters, we used CAT12 default settings. Using DARTEL (Ashburner, 2007), images were spatially normalized to the default IXI555 MNI template with 121×145×121 voxels of 1.5 mm cubic size. Due to high accuracy of DARTEL registration, we chose a rather small Gaussian smoothing kernel size of 4 mm. To correct for different head sizes, each subject's image was intensity normalized to its TIV. Only voxels within the SPM intracranial mask (mask_ICV.nii) were included for subsequent analysis. All analyses were performed with MATLAB 2020a (MathWorks, Sherborn, MA, USA), with parallel processing on up to 20 CPUs.

### 2.5 Calculating read‐out patterns

Read‐out patterns were calculated as difference of mean images between patients and HC from the same database. When planning the simulation, we decided to calculate the read‐out patterns from balanced samples, although this is not necessary. With the exception of PPMI, all databases provided more HC than patients. Therefore, randomly chosen HC subsamples were used for calculating the read‐out patterns. For PD analysis, we had more patients than HC, thus, PD patients with largest MoCA scores (corresponding to no and very mild cognitive impairment) were excluded from analysis (remaining PD patients had MoCA ≤ 28). With the exception of MDD, mean age differences between patients and HC were ≤ 2 years, MDD patients were 3.1 years older than HC. To investigate the effect of uncertainty in the read‐out pattern, we also calculated an AD pattern from a small subsample of 30 HC versus 30 patients (Monte–Carlo analysis, 30 repetitions).

### 2.6 Calculating NPV patterns

The PCA sample comprised HC from all databases but the one from where subjects were taken for read‐out pattern calculation and classification. We then selected “loosely age‐matched” HC for PCA within the age range of read‐out pattern subjects ±5 years. Due to the large PCA sample sizes (see Table 2) and, particularly, due to the large number of voxels, calculation of the voxel by voxel covariance matrix was too computationally demanding. Instead, we obtained NPV patterns via the smaller subject–subject covariance matrix (Bishop, 2006). We implemented this workaround as MATLAB‐script fastpca.m and made it available on the MathWorks servers.

### 2.7 Effect of PCA sample size on NBe

To investigate how the PCA sample size affects NBe, we performed PCA with random subsamples of the PCA sample (*n* = 20, 40, 100, 200, 300 out of 683, Monte–Carlo simulation with 20 repetitions). For each Monte–Carlo run, NBe was calculated after denoising with 1 to *n* PCs. The read‐out pattern was generated from all 205 AD versus 205 HC, the same 205 HC were used as residuals in NBe, R in Equation (12).

### 2.8 Comparing NBe and *t*‐ratio for different weights

To investigate the ability of the NBe to predict classification improvement, we compared the NBe with the *t*‐ratio, defined as *t*‐value after CODE divided by *t*‐value before CODE (two‐sample *t*‐test of PES, patients vs. HC). Both *t*‐ratio and NBe correspond to the factor by which the signal‐to‐noise ratio improves after denoising, with the difference that the *t*‐ratio also depends on the variance of the degree of pathological alterations in patients. In addition to the AD pattern described above (205 AD vs. 205 HC), we calculated noisier AD patterns from subsamples of 30 AD versus 30 HC. To illustrate the importance of correctly estimating the degree of uncertainty contained in the read‐out pattern, we calculated the NBe (Equation 9) twice, the second time with ${\mathrm{SE}}_{b}^{2}=0$ and ${\mathrm{SE}}_{b^{d}}^{2}=0$. For denoising, we varied *w* from 0 to 1, in order to demonstrate the ability of the NBe to find the optimal weight. Pattern expression scores were calculated in a leave‐one‐pair‐out fashion, that is, the very patient–HC pair for which the PES were calculated, had been excluded from read‐out pattern calculation. The analysis for the smaller subsamples was repeated 20 times with a new random subsample being drawn for each run.

### 2.9 Classification analyses

For each of the five read‐out patterns (AD vs. HC, PD vs. HC, SCZ vs. HC, AUT vs. HC and MDD vs. HC), we calculated NPV patterns from the associated PCA sample (see Table 2 and Section 2.6). The HC used for read‐out pattern calculation were also used as R for calculation of the estimation errors $\hat{\varepsilon }$, ${\hat{\varepsilon }}^{d}$, and NRe. Unless specified otherwise, calculation of optimal weights w (Equation 13 with Interior Point Optimization, (Byrd et al., 2000) and bounds 0≤w≤1), of CODE parameters NBe, SRe and NRe (according to 10) and of PES (three PES: before and after CODE and with SVM) was carried out in a leave‐one‐pair‐out (LOPO) fashion: with the exception of one subject pair (one patient, one HC, randomly paired), all subjects were used to calculate the read‐out pattern and to optimize NBe and to train and optimize a linear SVM (using standardized training data and bayesian optimization of the L2 regularization parameter lambda). PES were then calculated in the two remaining subjects. Optionally, analyses are repeated with w=1 (in results Tables 3 and 5 denoted as “CODE1”).

**TABLE 3:** CODE parameters for the read‐out pattern derived from Alzheimer's disease versus healthy controls (HC).

		AD vs. HC (large sample: *n* = 2 × 205), NCe of *b* = 4%	AD vs. HC (subsample *n* = 2 × 30), NCe of *b* = 24%	
Denoising method		CODE	CODE	CODE1
CODE parameters	*w*	0.9988	0.9454	1
	NRe (%)	98	92	95
	SRe (%)	96	91	95
	NBe	2.1	1.2	0.9
NCe of *b^d^* (%)		35	62	79

*Note*: To demonstrate the effect of sample size, sub‐samples (*n* = 2 × 30) were randomly drawn (20 repetitions) and mean values are reported.

Abbreviations: AD, Alzheimer's disease; *b^d^*, read‐out pattern after denoising; *b*, read‐out pattern; CODE1, CODE with optimization weight *w* = 1; NBe, net benefit estimator $\left(\mathrm{NBe}=\left(1-\mathrm{SRe}\right)/\left(1-\mathrm{NRe}\right)\right)$; NCe, noise component estimator; NRe, noise reduction estimator; SRe, signal reduction estimator.

**TABLE 4:** Analyses using the read‐out pattern derived from Alzheimer's disease (AD) versus healthy controls (HC).

	AD vs. HC			MCIc vs. HC			MCIc vs. MCIs			MCIc vs. MCIs			
Cross‐validation	Leave‐one‐pair‐out			Not necessary			Not necessary			Not necessary			
Classification method	PES	PES and CODE	SVM	PES	PES and CODE	SVM	PES	PES and CODE	SVM	PES	PES and CODE	PES and CODE1	SVM
*t*‐Value	15.8	26.2	21.7	9.5	20.9	17.6	7.0	9.5	9.7	7.0	8.2	7.4	8.1
AUC	0.86	0.96*	0.94	0.79	0.96*	0.92	0.75	0.81**	0.82	0.75	0.78***	0.76	0.77
SENS	0.75	0.92	0.84	0.71	0.96	0.89	0.79	0.79	0.75	0.76	0.77	0.74	0.74
SPEC	0.84	0.91	0.89	0.76	1.00	0.83	0.62	0.71	0.78	0.65	0.70	0.68	0.70

*Note*: In MCIc versus HC analysis, 151 age‐matched HC were removed from PCA sample and used for testing. One‐sided *p*‐values for comparison of AUC before and after CODE.

Abbreviations: CODE, controls‐based denoising; CODE1, CODE with weights *w* = 1; MCIc, mild cognitive impairment with conversion to AD in 6 years follow‐up; MCIs, stable MCI; PES, pattern expression score; SVM, linear support‐vector machine; *w*, optimization weight.

* *p* < .001;

** *p* = .01;

*** *p* < .02.

**TABLE 5:** Analyses of the other patient groups.

		PD vs. HC, NCe of *b* = 55%				AUT vs. HC, NCe of *b* = 69%			SCZ vs. HC, NCe of *b* = 13%			MDD vs. HC, NCe of *b* = 88%		
Classification method		PES	PES and CODE1	PES and CODE1, age pattern removed	SVM	PES	PES and CODE	SVM	PES	PES and CODE	SVM	PES	PES and CODE	SVM
CODE parameters	*w*	—	1.0	1.0	—	—	0.99	—	—	1.0	—	—	1.0	—
	NRe (%)	—	98	13	—	—	78	—	—	93	—	—	92	—
	SRe (%)	—	99	16	—	—	65	—	—	87	—	—	67	—
	NBe	—	0.3	1.0	—	—	1.6	—	—	2.0	—	—	4.5	—
NCe of *b^d^* (%)		—	98	42	—	—	76	—	—	30	—	—	92	—
Classification results (Leave‐one‐pair‐out)	*t*‐Value	2.4	0.6	2.4	0.7	3.9	7.1	6.9	15.4	35.1	22.4	0.2	3.0	0.6
	AUC	0.60	0.54	0.60	0.48	0.60	0.66*	0.53	0.92	1.00*	0.98	0.50	0.62**	0.53
	SENS	0.61	0.54	0.53	0.57	0.64	0.69	0.60	0.88	0.98	0.95	0.40	0.57	0.67
	SPEC	0.60	0.54	0.71	0.53	0.56	0.57	0.62	0.90	0.98	0.91	0.73	0.67	0.63

*Note*: We therefore do not separately report CODE with optimal weight. PD versus HC analysis was repeated after removing an age‐related pattern from PCA subjects and thus from NPV patterns. One‐sided *p*‐values for comparison of AUC before and after CODE. In PD versus HC, optimal weight was *w* = $10^{-7}$, equivalent to not performing CODE.

Abbreviations: AUT, autism; *b^d^*, read‐out pattern after denoising; *b*, read‐out pattern; CODE, controls‐based denoising; CODE1 no age, CODE after removing an age pattern; CODE1, CODE with weights *w* = 1; MDD, major depressive disorder. PES, pattern expression score; NBe, net benefit estimator $\left(\mathrm{NBe}=\left(1-\mathrm{SRe}\right)/\left(1-\mathrm{NRe}\right)\right)$; NCe, noise component estimator; NRe, noise reduction estimator; PD, Parkinson's disease; SCZ, Schizophrenia; SRe, signal reduction estimator; SVM, linear support‐vector machine.

* *p* < .001;

** *p* = .03.

For analyses of MCIc versus MCIs and MCIc versus HC, we used the AD read‐out pattern, therefore no cross‐validation was necessary. A previous exploratory analysis in PDD (different database, not shown) provided evidence that our PD read‐out pattern shares strong similarities with a VBM aging pattern. Therefore, we calculated an aging pattern by voxelwise regressing HC subjects (PCA sample) on their ages. This aging pattern was then removed from PCA subjects before calculating NPV patterns. This has the effect that all PCs were orthogonal to the aging pattern.

PES classification performances were evaluated with two‐sample *t*‐test and ROC analysis. Increase in AUC was tested for significance using the method of DeLong (DeLong et al., 1988; Sun & Xu, 2014), one‐sided *p*‐values are reported. Sensitivity and specificity were defined according to the Youden's index (Schisterman et al., 2005; Youden, 1950). Mean values over LOPO runs are reported.

## 3 RESULTS

### 3.1 Effect of PCA sample size on NBe

**FIGURE 2:**
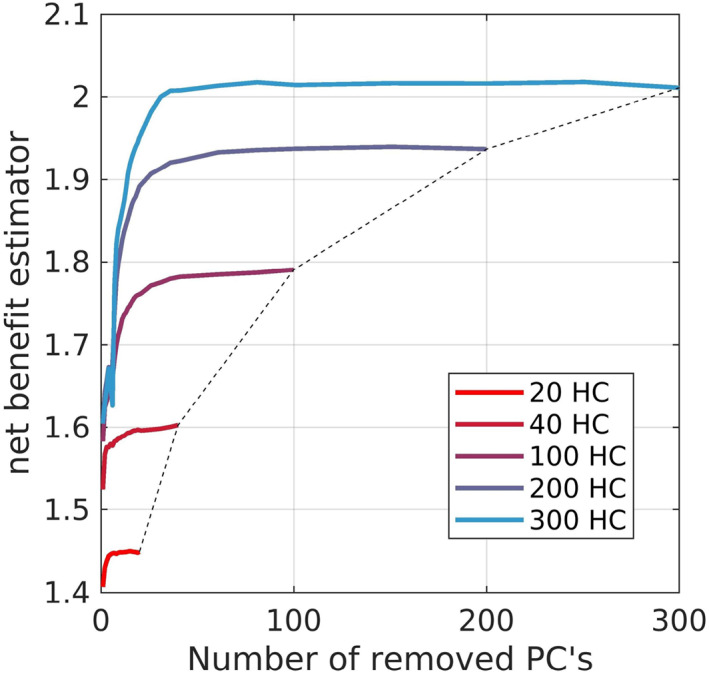
Impact of PCA sample size on the net benefit estimator (NBe, mean values from 20 Monte–Carlo runs) for the AD read‐out pattern.

Mean NBe as a function of the PCA sample size and of the number of removed PC's are shown in Figure 2. All curves reached a plateau after removing 10%–50% of all available PCs while increasing the PCA sample size still improved the NBe, corresponding to the fact that PCs from larger samples are more generalizable.

### 3.2 Comparing NBe and *t*‐ratio for different weights

**FIGURE 3:**
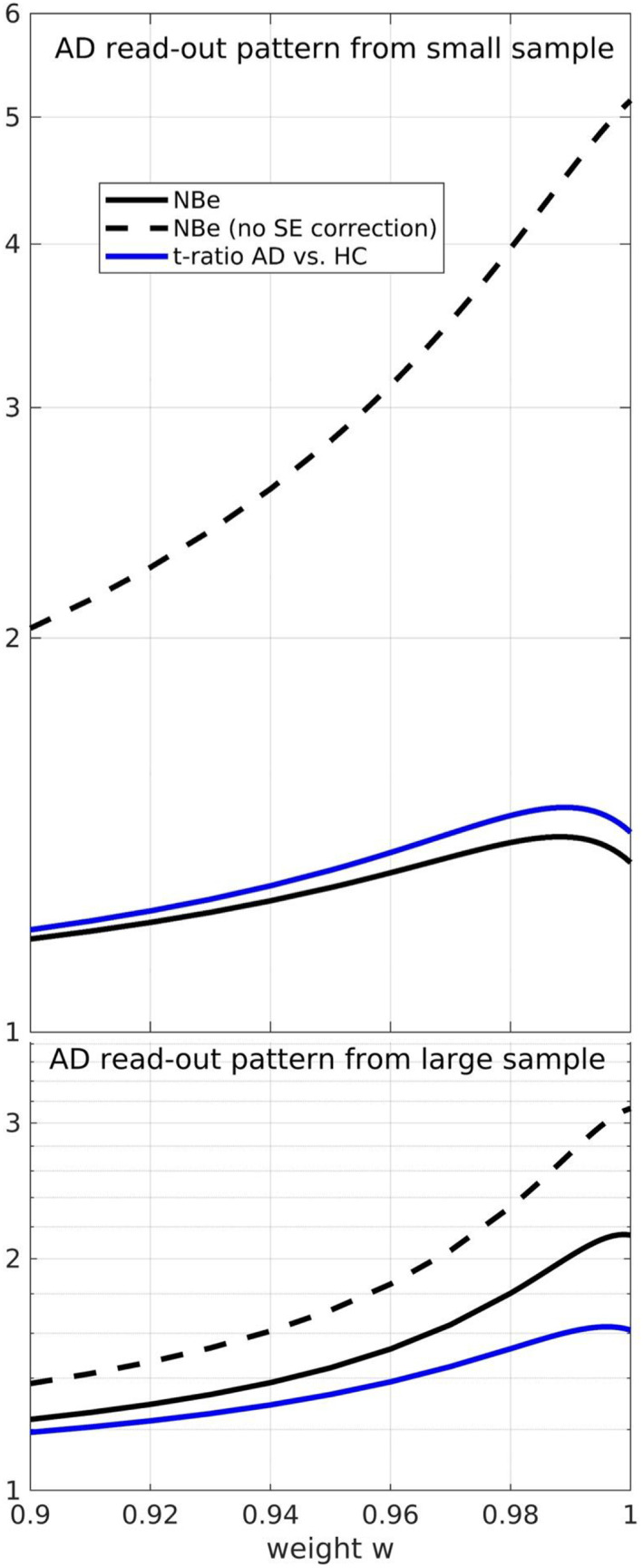
NBe and *t*‐ratio (Alzheimer's disease [AD] vs. healthy controls) for weighted denoising and two read‐out patterns with different levels of uncertainty. To illustrate the importance of uncertainty correction of NBe, we also depicted the uncorrected NBe.

Figure 3 shows NBe and t‐ratio (AD vs. HC) as a function of weights for weighted denoising and two read‐out patterns (top: small sample, that is, high level of uncertainty, bottom: large sample, i.e., low level of uncertainty). To illustrate the importance of uncertainty correction of NBe, we also depicted the uncorrected NBe. Albeit the *t*‐ratio differs from NBe by accounting also for pathological variance, NBe was found to correspond well with the *t*‐ratio, in particular in terms of the location of their maxima. For exploratory reasons, we investigated how NBe for the AD read‐out pattern is affected by PCA sample size (results are given in the supplementary material).

### 3.3 CODE parameters and classification analyses

CODE parameters and results from classification analyses are shown in Tables 3, 4, 5. According to the pattern uncertainty index NCe, read‐out patterns for AD and SCZ were the most stable ones (NCe = 4% and 13%), in contrast to PD, autism, and MDD (NCe = 55%, 69% and 90%). For the AD read‐out pattern (large sample), NBe was 2.1 and, accordingly, all classification analyses were significantly improved by CODE, that is, AD versus HC (AUC 0.96 vs. 0.86, *p* < .001), MCIc versus MCIs (AUC 0.81 vs. 0.75, *p* = .01) and MCIc versus HC (AUC 0.96 vs. 0.79, *p* < .001). Interestingly, the mean optimal weight was approximately 1.0 (*w* = 0.9988). When calculating the read‐out pattern from a small subsample (*n* = 2 × 30, NCe = 89%), classification by PES alone yielded results similar to those obtained with the large sample, however, CODE was less successful (NBe 1.2 vs. 2.1, corresponding to AUC 0.78 vs. 0.81) even with optimized weight. Interestingly, the rather small difference between the optimal weight (*w* = 0.9454) and standard weight (*w* = 1) corresponded to a noticeable difference in NBe and classification performance. Across LOPO‐runs, w, NBe, SRe, and NRe showed negligible variation (<1%). With the exception of PD versus HC, the remaining analyses also could significantly be improved by CODE. In particular, MDD versus HC had a very large median NBe of 4.5 but also a large variation across Monte–Carlo runs (from 2 to 12). The large NBe corresponds to the fact that, in MDD versus HC, both groups were hardly distinguishable before CODE (AUC = 0.5, *t* = 0.2).

**FIGURE 4:**
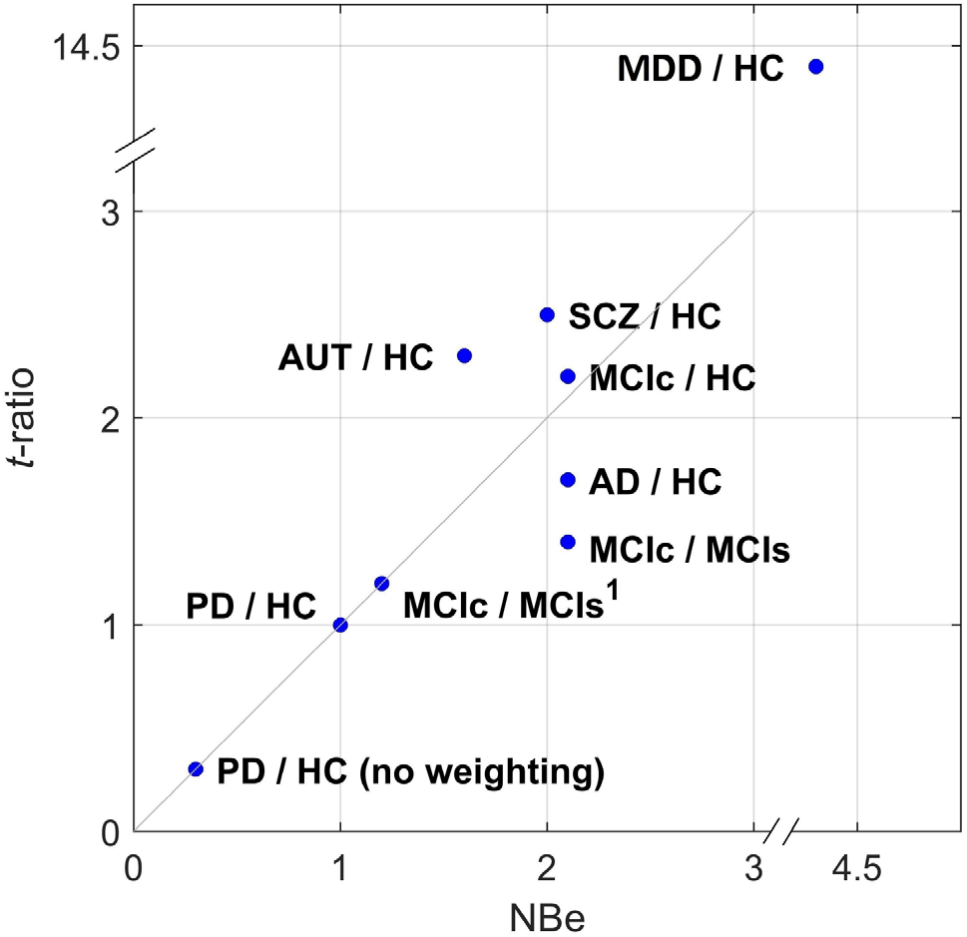
Predicted and actual classification improvement. ^1^With the AD read‐out pattern being calculated from a small subsample.

PD versus HC analysis was the only analysis that could not be improved by CODE. Accordingly, the optimizer found *w* = 0. Applying standard weights *w* = 1, CODE1 reduced classification performance, which was correctly predicted by NBe (NBe = 0.3 and AUC 0.54 vs. 0.60). Removing the aging‐pattern from PCA controls increased NBe to 1.0 for denoising with weight 1 and reduced SRe from 99% to 13%, which confirmed our hypothesis that the poor performance of CODE in this analysis was due to high similarity between the aging patten and the PD read‐out pattern. To give an overview of how good predicted and actual classification performance (*t*‐ratio vs. NBe) correspond to each other, the results from Tables 3 and 5 are shown in a scatter plot (Figure 4).

As compared to SVM, the presented method generally yielded better classification performances, most notably in MDD versus HC (*t* = 3.0 vs. 0.6, AUC 0.62 vs. 0.53) and MCIc vs HC (*t* = 20.9 vs. 17.6, AUC 0.96 vs. 0.92) but also in PD versus HC (*t* = 2.4 vs. 0.7, AUC 0.60 vs. 0.48).

For exploratory reasons, we repeated all classification without TIV‐normalization and generally obtained larger net benefits of CODE (higher NBe, higher *t*‐ratio and larger classification improvements due to worse pre‐CODE results, data not shown).

## 4 DISCUSSION

In quantifications based on PES, uncertainty contained in the read‐out pattern propagates into PES. We here propose the concept of squared standard error of a random pattern, ${\mathrm{SE}}_{b}^{2}$, in order to quantify the degree of uncertainty in a read‐out pattern. Using ${\mathrm{SE}}_{b}^{2}$, it is possible to more accurately estimate the error when using a pattern expression score (PES) to estimate alterations caused by pathology. We applied this concept to controls‐based denoising (CODE) and were able to predict the net benefit of CODE in terms of the expected improvement of the signal‐to‐noise ratio (NBe). Furthermore, with an improved estimator of the net benefit, we were able to optimize the weights used for denoising. With weighted CODE, we were able to significantly improve classifications based on voxel‐based morphometry patterns in the most common neurodegenerative and neuropsychiatric disorders. The importance of an uncertainty correction was most evident for read‐out patterns derived from a small training sample and in diseases where alterations caused by pathology are small as compared to physiological variance (e.g., Parkinson's disease).

In the view of a fast increasing number of machine learning algorithms, CODE stands out by (i) taking information from a separate control sample that does not have to be perfectly matched and thus can be taken from a multi‐center database, (ii) the fact that only a rather small number of patients is needed to define the read‐out pattern, and (iii) simplicity. We suggest using CODE as a complement to PES‐based quantification for both clinical and research purpose, for classification as well as for advanced assessment of biomarkers.

The benefit of denoising, that is, reduced variance from various sources not related to pathology (here denoted estimated noise reduction, NRe) has to be weighed against the cost of denoising (here denoted estimated signal reduction SRe), that is, a removal of pathology‐related changes that bear resemblance with one or multiple patterns of nonpathological variance. In CODE, it is conceivable that denoising does more harm than good, therefore it is crucial to accurately predict the effect of denoising. When using an optimized weight as suggested, it can be ruled out that CODE does more harm than good and the worst case would be that CODE has no effects on the PES.

The PES is a well‐established tool for classification (Blazhenets et al., 2020; Blazhenets et al., 2021; Meles et al., 2018; Oh et al., 2014; Sörensen et al., 2020). With CODE, we improved all classifications with the exception of PD versus healthy controls (see below for a discussion of the findings in PD). We achieved better performances of AD conversion prediction than most of those reported in the literature (Beheshti et al., 2017; Moradi et al., 2015; Salvatore et al., 2015; Salvatore et al., 2016), despite the fact that, in the literature, clinical follow‐ups were considerably shorter (<3 years, in contrast to the maximum follow‐up time of 6 years used in our analysis), excluding converters with an early stage of AD pathology and thus facilitating discrimination from nonconverters. A similar classification performance (AUC = 0.81) than ours was reported using deep learning (Abrol et al., 2018). In the literature, MCIc has also been compared to HC. Comparing MCIc to HC, again, our classification performance was superior or in the same order of magnitude as compared to the literature (Cao et al., 2020; Gupta et al., 2019).

While one might consider our perfect classification result between schizophrenia and HC to be suspicious, very high AUC can also be found in the literature (Liu et al., 2017).

Our classification result between MDD versus HC (AUC = 0.62) is comparable to the best results from the Predictive Analytics Competition (PAC) 2018, where the winner team achieved 65% balanced accuracy (Gao et al., 2018).

In PD, CODE considerably worsened classification results, which was correctly predicted by the NBe (<1.0). The reason for this was that the PD‐pattern (the square of its Euclidean length) was reduced by more than 99% (SRe) when removing patterns of nonpathological variance. Based on observations from prior studies, we hypothesized that the problem was the high degree of similarity between the PD pattern and the pattern of age‐related changes. We were able to confirm this hypothesis by removing age‐related changes from the control subjects that were used for PCA. As a consequence, CODE no longer removed this pattern from patients and the SRe dropped from 99% to 16%.

For research and clinical application of CODE, it is crucial to recognize the situation where the cost of CODE is higher than the benefit as, for example, in our analysis of PD patients. The NBe did mirror this situation, however, only after including the presented correction term, accounting for the level of uncertainty in the read‐out pattern.

## 5 LIMITATIONS

Pattern expression score is a linear concept with all pros and cons of linearity. The most important disadvantage is that pathological alterations do not necessarily progress in a linear fashion on a voxel‐level. The advantage is the transparency and simplicity, yielding a robust biomarker easy to interpret. Furthermore, in this article, we showed that a linear pathology model allows for a straightforward quantification of the implications of read‐out pattern uncertainty. Due to the linearity of PES and CODE, we chose linear SVM with optimized L2‐regularization strength for comparison while, obviously, more sophisticated machine learning algorithms (e.g., nonlinear SVM, deep learning) might yield better classification performance. After all, a comparison between optimized CODE and optimized SVM was not the primary goal of this article and the respective AUC differences were not tested for statistical significance. Nonlinear alternatives for calculation of NPV patterns (e.g., nonlinear PCA) seem feasible but where considered beyond the scope of this work. Finally, we want to point out that some of the patients from ABIDE database used for validation were children, for which the stereotactic normalization procedure has not been adjusted (no age dependent tissue probability map) so that, hypothetically, VBM may allow for an even better classification between patients with autism and healthy subjects.

## 6 CONCLUSION

We conclude that the degree of uncertainty in a read‐out pattern can be quantified within the presented theoretical framework and should generally be reported in PES‐based analyses, ideally in terms of the estimated normalized ${\mathrm{SE}}^{2}$, here denoted noise component estimator NCe. The usefulness of the proposed measure was demonstrated by optimizing CODE for quantification of pathological changes in VBM in the most common neurodegenerative and neuropsychiatric diseases. CODE stands out by using information from a control sample that is only loosely matched and that is easily available from multicenter databases. To find optimal weights for CODE and to avoid a possible worsening of classification performance, accounting for the degree of uncertainty in the read‐out pattern is necessary. We suggest using weighted CODE as a complement to PES‐based quantification for both research and clinical purpose, for classification as well as for advanced assessment of biomarkers.

## FUNDING INFORMATION

This research received no specific grant from any funding agency in the public, commercial, or not‐for‐profit sectors.

## CONFLICT OF INTEREST STATEMENT

Dominik Blum declares that he has no conflict of interest. Tobias Hepp declares that he has no conflict of interest. Vladimir Belov declares that he has no conflict of interest. Roberto Goya‐Maldonado declares that he has no conflict of interest. Christian la Fougére declares that he has no conflict of interest. Matthias Reimold declares that he has no conflict of interest.

## ETHICS STATEMENT

The Ethics Committee of the University Medical Center Göttingen (UMG) approved the study protocols (EK 24/8/18, EK 25/7/16, EK 27/10/15) and all subjects provided their informed consent before investigation. Except by the UMG, this article does not contain any studies with human participants performed by the authors.

## Supporting information


**Data S1.** Supporting Information.

## Data Availability

ABIDE: Autism Brain Imaging Data Exchange II, http://fcon_1000.projects.nitrc.org/indi/abide/abide_II.html. ADNI: Alzheimer's Disease Neuroimaging Initiative database (adni.loni.usc.edu). A complete listing of ADNI investigators can be found at: http://adni.loni.usc.edu/wp-content/uploads/howtoapply/ADNIAcknowledgementList.pdf. AIBL: Australian Imaging Biomarkers and Lifestyle flagship study of ageing funded by the Commonwealth Scientific and Industrial Research Organisation (CSIRO) which was made available at the ADNI database www.loni.usc.edu/ADNI. AIBL researchers are listed at www.aibl.csiro.au. COBRE & FBIRN: The Function Biomedical Informatics Research Network Data Repository (FBIRN) and International Neuroimaging Data‐sharing Initiative (COBRE) via http://schizconnect.org. IXI: Information eXtraction from Images (EPSRC GR/S21533/02). This data is made available under the Creative Commons CC BY‐SA 3.0 license. PPMI: Data used in the preparation of this article were obtained from the Parkinson's Progression Markers Initiative (PPMI) database www.ppmi-info.org/data.

## APPENDIX A

This appendix supplements Section 2.2.1, that is, the theory related to the linear pathology model, the PES and the PES error in the presence of a deviation between b and **μ**.

The PES of *μ* is defined as

(A1)
$${\mathrm{PES}}_{\mu ,i}=\frac{\left\langle p_{i},\mu \right\rangle }{{\left\|\mu \right\|}^{2}}$$

and, within the framework of the linear pathology model, can be used to estimate ${\mathrm{PSS}}_{i}$ in terms of

(A2)
$${\mathrm{PES}}_{\mu ,i}={\mathrm{PSS}}_{i}+\epsilon_{i}={\hat{\mathrm{PSS}}}_{i}.$$

with the error $\epsilon_{i}$ being given by

(A3)
$$\epsilon_{i}=\left\langle r_{i},\mu \right\rangle /{\left\|\mu \right\|}^{2}.$$

When b is used instead of **μ**, the PES, given by Equation (2), is expected to be proportional to ${\mathrm{PSS}}_{i}$:

(A4)
$${\mathrm{PES}}_{i}\propto \frac{\left\langle p_{i},b\right\rangle }{\left\langle \mu ,b\right\rangle }={\mathrm{PSS}}_{i}+\epsilon_{i}={\hat{\mathrm{PSS}}}_{i}$$

with

(A5)
$$\epsilon_{i}=\left\langle r_{i},b\right\rangle /\left\langle \mu ,b\right\rangle .$$

The factor of proportionality $\left\langle \mu ,b\right\rangle /{\left\|b\right\|}^{2}$ is unknown, but can be neglected insofar as it is the same for all subjects and therefore does not affect classification based on PES.

In this article, we calculate b as mean difference between patients P and healthy controls H,

(A6)
$$b=\bar{P}-\bar{H}.$$

For finite samples $\left(P,H\right)$, there is a random deviation Δb=b−μ. We expect b to represent the unknown true pathological pattern **μ**, thus $E\left[b\right]=\mu$ and therefore $E\left[\Delta b\right]=0$. As **μ** is unknown, $\epsilon_{i}$ cannot be calculated directly. In order to estimate $\epsilon_{i}$, we first rewrite $\left\langle \mu ,b\right\rangle$ in terms of the squared Euclidean length of Δb, that is, ${\left\|\Delta b\right\|}^{2}$, a measure that can be easily estimated as shown below.

(A7)
$$\left\langle \mu ,b\right\rangle ={\left\|b\right\|}^{2}-\left({\left\|\Delta b\right\|}^{2}+\left\langle \mu ,\Delta b\right\rangle \right).$$

We then estimate $\left\langle \mu ,b\right\rangle$ by replacing ${\left\|\Delta b\right\|}^{2}+\left\langle \mu ,\Delta b\right\rangle$ with its expected value. $E\left[\left\langle \mu ,\Delta b\right\rangle \right]$ vanishes and $E\left[{\left\|\Delta b\right\|}^{2}\right]=E\left[{\left\|b-\mu \right\|}^{2}\right]$ remains, that is, the expected squared Euclidean distance between a random pattern b and the population mean **μ**, thus

(A8)
$$E\left[\left\langle \mu ,b\right\rangle \right]={\left\|b\right\|}^{2}-E\left[{\left\|b-\mu \right\|}^{2}\right].$$

$E\left[{\left\|b-\mu \right\|}^{2}\right]$ corresponds to the definition of the variance of a scalar random variable x, $\mathrm{Var}\left(x\right)=E\left[{\left(x-E\left[x\right]\right)}^{2}\right]$, with the only difference that b is a J‐dimensional vector (J being the number of voxels). According to this analogy, one might thus denote $E\left[{\left\|b-\mu \right\|}^{2}\right]$ the variance of b. However, this would be misleading insofar as the variance of a multivariate random variable such as b is usually defined as variance–covariance matrix and not as a scalar. Therefore, and due to the fact that the standard deviation of a sample statistic is often referred to as its standard error, we propose to denote $E\left[{\left\|b-\mu \right\|}^{2}\right]$ the *squared standard error* of b, ${\mathrm{SE}}_{b}^{2}$:

(A9)
$$\begin{array}{l} {\mathrm{SE}}_{b}^{2}=E\left[{\left\|b-\mu \right\|}^{2}\right]=E\left[\sum_{j=1}^{J}{\left(b_{j}-\mu_{j}\right)}^{2}\right]\\ =\sum_{j=1}^{J}\left(E{\left[b_{j}-\mu_{j}\right]}^{2}\right)=\sum_{j=1}^{J}\mathrm{Var}\left(b_{j}\right). \end{array}$$

In order to estimate ${\mathrm{SE}}_{b}^{2}$ from our data sample $\left(P,H\right)$, we recall that $\mathrm{Var}\left(b\right)=\mathrm{Var}\left(\bar{P}-\bar{H}\right)=\mathrm{Var}\left(\bar{P}\right)+\mathrm{Var}\left(\bar{H}\right)$ because $\bar{P}$ and $\bar{H}$ are independent. Assuming Gaussian distribution for P and H, the variance of mean is given by the variance of the sample divided by its size. Thus, the estimator of ${\mathrm{SE}}_{b}^{2}$ is given by:

(A10)
$$\begin{array}{l} {\mathrm{SE}}_{b}^{2}=\frac{1}{N_{P}\left(N_{P}-1\right)}\sum_{i\in I,j\in J}{\left(P_{i,j}-{\bar{P}}_{j}\right)}^{2}\\ +\frac{1}{N_{h}\left(N_{h}-1\right)}\sum_{i\in I,j\in J}{\left(H_{i,j}-{\bar{H}}_{j}\right)}^{2}. \end{array}$$
