# Supplementary material for: Estimating uncertainty in read‐out patterns: Application to controls‐based denoising and voxel‐based morphometry patterns in neurodegenerative and neuropsychiatric diseases
Source: Hum Brain Mapp. 2023 Mar 22;44(7):2802–14. doi: 10.1002/hbm.26246 (PMC10089107; doi:10.1002/hbm.26246)
Supplement: Supplementary file 1 — Data S1. Supporting Information. [file HBM-44-2802-s001.pdf]

Supplementary material for: Estimating uncertainty in read-out patterns: application to controls-based denoising and voxel-based morphometry patterns in neurodegenerative and neuropsychiatric diseases

Dominik Blum, Tobias Hepp, Valdimir Belov, Roberto Goya-Maldonaldo, Christian la Fougère and Matthias Reimold

A closed-form solution for weighted denoising

We derive the analytical solution for optimal NPV-pattern weights, with the assumption that the read-out pattern $\mathbf{b}$ equals the true pathological pattern $\boldsymbol{\mu}$, i.e. $\mathrm{SE}_{\mathbf{b}}^2 = 0$. According to eq. 13, the objective function then reduces to

$$\mathbf{w}^* = \mathrm{argmin}_{\mathbf{w}} \sigma\left(\hat{\boldsymbol{\epsilon}}^d\right) = \mathrm{argmin}_{\mathbf{w}} \frac{\sigma\left(\mathbf{R}\mathbf{b}_{\mathbf{w}}^{d\,\intercal}\right)}{|\langle \mathbf{b}, \mathbf{b}_{\mathbf{w}}^d \rangle|}. \tag{1}$$

As we here allow for different weights for each PC, the read-out pattern after denoising is $\mathbf{b}_{\mathbf{w}}^d = \mathbf{b} - \mathbf{w}^\intercal \mathbf{C}^\intercal \mathbf{C}$ with the solution column vector $\mathbf{w}$. For convenience, we redefine constants to $\boldsymbol{\alpha}_1 = \mathbf{R}\mathbf{b}^\intercal, \boldsymbol{\alpha}_2 = \mathbf{R}\mathbf{C}^\intercal \mathrm{diag}(\mathbf{b}\mathbf{C}^\intercal), \alpha_3 = \|\mathbf{b}\|^2$ and $\boldsymbol{\alpha}_4 = \mathbf{b}\mathbf{C}^\intercal \mathrm{diag}(\mathbf{b}\mathbf{C}^\intercal)$, i.e.

$$\mathbf{w}^* = \mathrm{argmin}_{\mathbf{w}} \frac{\|\boldsymbol{\alpha}_1 - \boldsymbol{\alpha}_2 \mathbf{w}\|^2}{(\alpha_3 - \boldsymbol{\alpha}_4 \mathbf{w})^2}. \tag{2}$$

Differentiating eq. 2 with respect to $\mathbf{w}$ and setting to zero gives

$$\mathbf{w} = \left(\boldsymbol{\alpha}_2^\intercal \boldsymbol{\alpha}_2\right)^{-1} \boldsymbol{\alpha}_2^\intercal \boldsymbol{\alpha}_1 - \frac{\|\boldsymbol{\alpha}_1 - \boldsymbol{\alpha}_2 \mathbf{w}\|^2}{\alpha_3 - \boldsymbol{\alpha}_4 \mathbf{w}} (\boldsymbol{\alpha}_2^\intercal \boldsymbol{\alpha}_2)^{-1} \boldsymbol{\alpha}_4^\intercal. \tag{3}$$

In the following, the normal equation $\left(\boldsymbol{\alpha}_2^\intercal \boldsymbol{\alpha}_2\right)^{-1} \boldsymbol{\alpha}_2^\intercal \boldsymbol{\alpha}_1$ is denoted $\mathbf{w}_0$, the ratio $\|\boldsymbol{\alpha}_1 - \boldsymbol{\alpha}_2 \mathbf{w}\|^2 / (\alpha_3 - \boldsymbol{\alpha}_4 \mathbf{w})$ is denoted (scalar) t and $\boldsymbol{\beta} = (\boldsymbol{\alpha}_2^\intercal \boldsymbol{\alpha}_2)^{-1} \boldsymbol{\alpha}_4^\intercal$. Only t is unknown and can be formulated by replacing $\mathbf{w}$ with $\mathbf{w}_0 - \mathrm{t}\boldsymbol{\beta}$:

$$t = \frac{\|\boldsymbol{\alpha}_1 - \boldsymbol{\alpha}_2 \mathbf{w}\|^2}{\alpha_3 - \boldsymbol{\alpha}_4 \mathbf{w}} = \frac{\|\boldsymbol{\alpha}_1 - \boldsymbol{\alpha}_2 \mathbf{w}_0 + t \boldsymbol{\alpha}_2 \boldsymbol{\beta}\|^2}{\alpha_3 - \boldsymbol{\alpha}_4 \mathbf{w} + t \boldsymbol{\alpha}_4 \boldsymbol{\beta}}. \tag{4}$$

Solving for t gives $\mathrm{t}(\alpha_3 - \boldsymbol{\alpha}_4 \mathbf{w}_0) + \mathrm{t}^2 \boldsymbol{\alpha}_4 \boldsymbol{\beta} = \mathrm{t}^2 \|\boldsymbol{\alpha}_2 \boldsymbol{\beta}\|^2 + 2\mathrm{t}(\boldsymbol{\alpha}_1 - \boldsymbol{\alpha}_2 \mathbf{w})^\intercal \boldsymbol{\alpha}_2 \boldsymbol{\beta} + \|\boldsymbol{\alpha}_1 - \boldsymbol{\alpha}_2 \mathbf{w}_0\|^2$, which can be reduced to $\mathrm{t}(\alpha_3 - \boldsymbol{\alpha}_4 \mathbf{w}_0) = \|\boldsymbol{\alpha}_1 - \boldsymbol{\alpha}_2 \mathbf{w}_0\|^2$, i.e.

$$\mathrm{t} = \frac{\|\boldsymbol{\alpha}_1 - \boldsymbol{\alpha}_2 \mathbf{w}_0\|^2}{\alpha_3 - \boldsymbol{\alpha}_4 \mathbf{w}_0}. \tag{5}$$

As t is now a function of $\mathbf{w}_0$, we can give the analytical solution for optimal NPV-pattern weights (with the assumption $\mathrm{SE}_{\mathbf{b}}^2 = 0$),

$$\mathbf{w}^* = \mathbf{w}_0 - \frac{\|\boldsymbol{\alpha}_1 - \boldsymbol{\alpha}_2 \mathbf{w}_0\|^2}{\alpha_3 - \boldsymbol{\alpha}_4 \mathbf{w}_0} (\boldsymbol{\alpha}_2^\intercal \boldsymbol{\alpha}_2)^{-1} \boldsymbol{\alpha}_4^\intercal. \tag{6}$$
